# Remanufacturing an evaluation system for electrical control systems of drilling rig based on the improved FCE and ANN

**DOI:** 10.1371/journal.pone.0268788

**Published:** 2022-05-25

**Authors:** Xinghua Dong, Zhiwei Zhang, Juan Sun, Zhen Luo

**Affiliations:** 1 School of Mechanical Engineering, Northwestern Polytechnical University, Xi ’an, Shanxi, China; 2 Department of Intelligent Control, CNPC National Engineering Research Center for Oil & Gas Drilling Equipment Co., LTD, Baoji, Shanxi, China; 3 CNPC National Engineering Research Center for Oil & Gas Drilling Equipment Co., LTD, Baoji, Shanxi, China; 4 Department of Standard Research, CNPC National Engineering Research Center for Oil & Gas Drilling Equipment Co., LTD, Baoji, Shanxi, China; 5 Department of Process Project,CNPC National Engineering Research Center for Oil & Gas Drilling Equipment Co., LTD, Baoji, Shanxi, China; University Campus Bio-Medico of Rome, ITALY

## Abstract

The decision process of different remanufacturing schemes in an electronic control system has great fuzziness and uncertainty. Therefore, it is essential to use an appropriate method to show the characteristics of different schemes and support the users’ decision. Based on the concepts of the artificial neural network theory and the improved comprehensive evaluation method, the decision-making system of the electronic control remanufacturing scheme was constructed in the present study. In the first step, a classification method of parts is proposed from the perspective of manufacturing enterprises. Moreover, an artificial neural network model is used to determine parts of remanufacturing value. Then the pricing strategy is divided according to the users’ needs, and then a decision model is constructed. The combined subjective and objective methods are used to solve the compound weight of different equipment, and a set of improved fuzzy comprehensive decision methods is formed. Then the proposed model was applied to an electronic control transformation project as an example to evaluate the performance of different schemes. The evaluation results were consistent with the results of a third-party organization. It was concluded that the proposed scheme can be used as the theoretical basis to choose the best remanufacturing scheme to ensure the efficient operation of each part in an ECS.

## Introduction

Oil drilling rig is a high-end strategic equipment in oil and gas exploration. An electronic control system (ECS) is an important part of the drilling rig, providing the main power and control equipment of the oil drilling rig [1]. ECS has been gradually developed in the petroleum industry since the end of the 20th century and has been widely used since then. At present, it is approaching the product overhaul cycle [2,3]. Remanufacturing is an effective method to prolong the life of electronic control systems where manufacturing enterprises are applied to formulate remanufacturing schemes of different configurations according to the state of recovered equipment. Although users can make decisions based on previous experience, this type of decision-making method needs a long-term accumulation of experience. Accordingly, there is no guarantee to use the optimal configuration as the selected remanufacturing scheme [4,5]. Meanwhile, since the expenses of electronic control systems for remanufacturing are higher than one million yuan, bad decisions will lead to economic losses. Moreover, it is a challenge to involve an ECS in electrical equipment and perform remanufacturing evaluations one by one. Consequently, the whole decision-making process has great fuzziness and uncertainty. In order to resolve these problems, manufacturing enterprises have been used to study the remanufacturing related policies, manufacturing strategies, and related decisions. In this regard, Jin Tian et al. [6] proposed a comprehensive evaluation method based on the concepts of gray and fuzzy processing to characterize the safety of the processing. Huilong Liu et al. [7] used the entropy weight fuzzy comprehensive evaluation method to evaluate the quality of short-cut carbon fiber in batch production. Hong-Wen li et al. [8] studied the ABC classification of petroleum production equipment based on the fuzzy analytic hierarchy process. Wanxiang Wang et al. [9] performed a grey relational analysis and showed that the fuzzy comprehensive judgment is an effective way to evaluate the service quality. In the reviewed investigations, the fuzziness of the evaluation indicator was used to establish the corresponding evaluation indicator system. Atalay et al. [10] showed that the key for users to choose remanufactured products is to evaluate their utility.

Although numerous investigations have been carried out on the decision-making and related treatment methods, the differences in user requirements have not yet been studied so far. This shortcoming mainly originates from uncertainties of the user demand. However, different users may use environmental differences, including the pursuit of the drilling speed, reducing the production cost, and ensuring the safety of the production process. Consequently, there may be different demands [11] for different remanufactured products [12–14], which affects the final decision. Recently, investigating different user demands has become a research direction affecting remanufacturing strategies. Dongyue Liu [15] deeply studied the role of association rules in data mining and practically improved these rules. Moreover, some special requirements put forward by users in the system-level remanufacturing should be satisfied. Aiming at viewing these two requirements, it is necessary to build a system-level remanufacturing evaluation system. Considering diverse remanufacturing schemes in different projects, equipment classification is usually carried out and then the results of different schemes are ranked and scored through evaluation. Meanwhile, it is of great significance to intuitively display the characteristics of each remanufacturing scheme to help users to make appropriate decisions according to their needs.

Based on the composition of the electronic control system, a classification method of parts is proposed using an ANN model. The main objective of the proposed model is to determine parts of remanufacturing value. Pricing strategy based on the user demand difference is divided and then a decision model is established using the subjective combining [16,17], objective combining [18,19], and weights combining [20,21] methods of different devices to form a set of improved fuzzy comprehensive decision [22–24]. The proposed scheme is expected to become an effective method to evaluate the characteristics of different schemes, support users to make a decision, and become a basis to optimize the designs.

## Analysis of the ECS

### Composition and internal correlation of ECS

ECS is a complex system consisting of several parts, including the power system, electric transmission drive automatic control system, and distribution control system [1].

### Classification of remanufactured parts

Because the electronic control system involves many parts, it is a great challenge to classify and make remanufacturing decisions one by one. By investigating ECS-related equipment enterprises and analyzing the technical indicators of each component of the system, a method is proposed to classify remanufactured parts from different aspects such as remanufacturing profit and overhaul cycle frequency. Accordingly, components can be mainly divided into three categories to reduce the classification time and speed up the classification:

1. Wearing parts: These parts are low value-added products and easy to damage, and have only one overhaul cycle. Springs, bolts, and cold-core are in this category.
2. Easy to wear: These parts have high remanufacturing profits, are easy to wear, and have multiple overhaul cycles. Examples are engine fuel injectors and fuel pumps.
3. Non-wearing parts: These parts have high remanufacturing profits, are not easily damaged, and have multiple overhaul cycles. Examples are electronic control modules.

The typical remanufacturing scheme and remanufacturing components of a single device are shown in Table 1.

**Table 1:** Typical remanufacturing equipment.

item	Subordinate to the system	Remanufacturing equipment	Remanufacturing Scheme	Remanufactured parts
1	Power system	1. Generator set [25]	Maintenance program: disassembly—cleaning—detecting—mechanical processing—test and painting	Engine parts, fuel systems, and so on
2	Electric drive automation control system	1. The main motor [26]	Maintenance program: disassembly—cleaning—detecting—testing and painting	Stator and rotor
			Modification scheme: disassembly—cleaning—partial replacement—detecting—testing and painting	Stator and rotor
		2. Electric control room [14]	Maintenance program: disassembly—cleaning—detecting—mechanical processing—test and painting	Room body structure parts
			Modification scheme: disassembly—cleaning—detecting—mechanical processing—adding auxiliary equipment—testing and painting	Room body structure parts
			Upgrade program: disassembly—cleaning—detecting—machining—adding main equipment—testing and painting	Room body structure parts
		3. Transformer [14]	Maintenance program: disassembly—cleaning—detecting—testing and painting	Transformer heat sink
3	Distribution control system	Auxiliary motor	Maintenance program: disassembly—cleaning—detecting—testing and painting	Stator and rotor
			Modification scheme: disassembly—cleaning—partial replacement—detecting—testing and painting	Stator and rotor

### Cost evaluation of component remanufacturing

**Fig 1:**
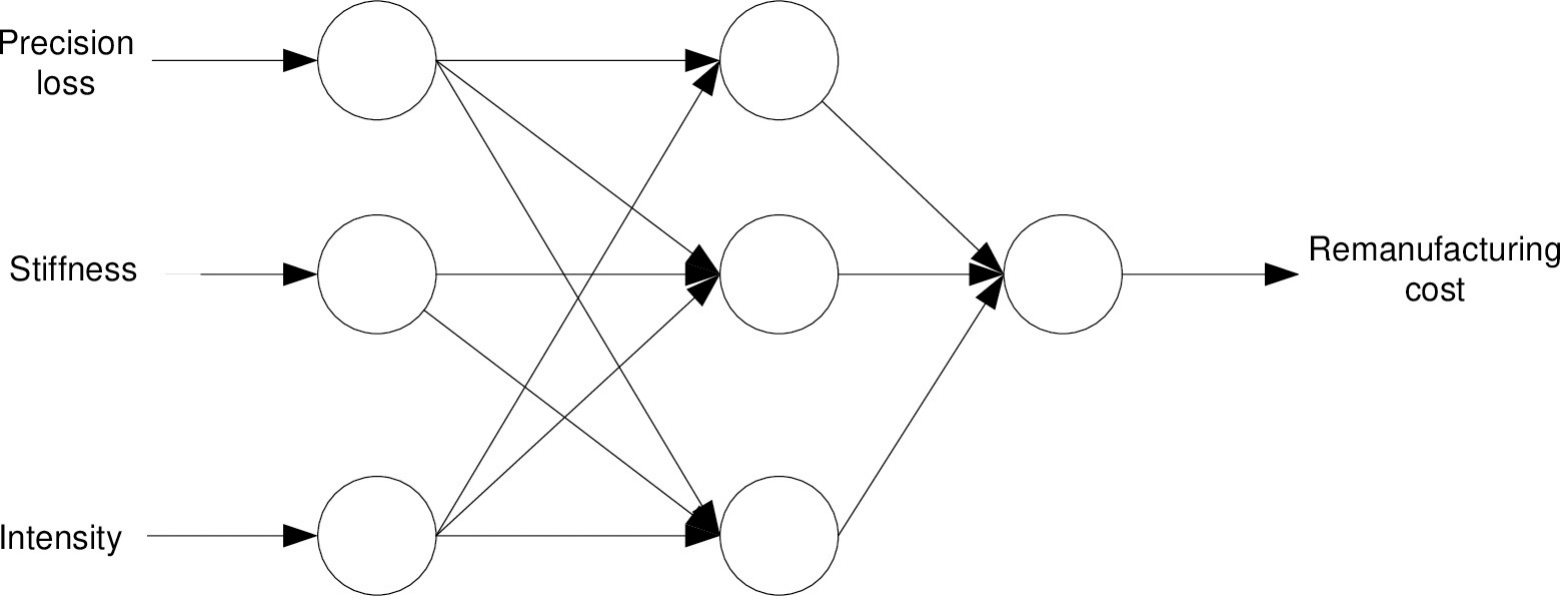
Spindle remanufacturing cost prediction model based on the ANN.

The diversity of failures of different parts makes the evaluation of remanufacturing costs uncertain and affects the selection of the appropriate remanufacturing method. Therefore, evaluation of part remanufacturing costs is of significant importance to determine which part has remanufacturing potential. Currently, the following three types of assessment methods are used in this respect: 1) mechanics-based assessment methods, including the nominal stress method [27] and the cumulative fatigue damage method [28]. 2) Evaluation methods based on finite element simulation, including dynamics simulation [29] and modal analysis [30]. 3) Assessment methods based on information technology, including ANN [31–33], deep learning method [34], and fuzzy calculation method [35]. For parts with a single failure, it is feasible to use the mechanics-based remanufacturing cost evaluation. However, in a real environment, parts often fail due to the joint action of multiple failure modes. Accordingly, this method has some limitations in real environments. In the evaluation method based on the finite element method, the prediction accuracy depends on the working conditions so the prediction results are uncertain. The cost evaluation method based on information technology is an ideal method, wherein the relationship between the data and remanufacturing cost is established based on the running status data of parts. It should be indicated that since the electronic control system is not fixed and is characterized by frequent moving, it is usually a challenge to query the historical working conditions of the parts disassembled from the electronic control system. In view of this situation, the ANN technology, which can learn and train itself to adapt to different information processing requirements, is usually used [27–29]. Further investigations reveal that the ANN technology is capable of dealing with problems with unclear background and inference rules. Meanwhile, it provides input characteristics of the ECS components that do not contain complex information such as images. Another advantage of the ANN technology compared with conventional technologies is that the input parameter is a few number instead of a large vector, thereby having less calculations. Accordingly, using the ANN can meet the requirements at this stage in the study of don’t need the deep learning technologies, unfold the other algorithms in the later research work. In the present study, the remanufacturing evaluation of parts is classified based on the above-mentioned classifications. The first type of parts is directly replaced, while a set of remanufacturing cost evaluation methods based on the ANN is proposed for the second and third types. Considering the spindle of the main motor remanufactured component [36] as the research object, the training, and testing process of ANN can be summarized as follows:

1. Nondestructive testing technology was used to extract the characteristic index of the spindle performance under different operating conditions.
2. Based on the ANN technology, the hyperparameters are the number of layers L in the neural network, the number of neurons J in each hidden layer, the learning rate η, and the number of iterations. In the present study, a single hidden layer (i.e. L = 1) with three neuron nodes (i.e. j = 3) was adopted. The performance degradation characteristic index of the remanufacturing spindle, including "precision loss", "stiffness" and "strength", was taken as the input for the neural network, and the corresponding "remanufacturing cost prediction value" was taken as the output. Moreover, the learning rate of the main motor spindle was set to η = 0.025. The number of iterations is determined according to the principle of "early stop". The accuracy rate stops after a period of time to determine the number of iterations. The prediction model of the relationship between performance degradation characteristic index and remanufacturing cost of the main motor spindle built through training is shown in Fig 1, and the training sample table is presented in Table 2.
3. When evaluating the cost of a similar main motor spindle in the later stage, the remanufacturing cost can be estimated only by inputting the characteristic index values into the model. The verification of the training effect of the neural network is presented in Table 3.
4. After estimating the remanufacturing cost, the model is analyzed. If the remanufacturing cost of the spindle is less than 70% (Experience value) of a new spindle, it has the value of reuse; otherwise, resource recovery is carried out.

**Table 2:** ANN training samples (part).

No	Precision loss (㎛)	Stiffness (N/㎛)	Intensity (MPa)	Remanufacturing cost(¥)
1	105	247.7	137	1870
2	131	201.7	179	1870
3	145	230.8	147	1870
4	149	197.7	142	1870
5	228	188.4	136	4290
6	279	243.3	189	4290
7	311	181.1	161	4290
8	328	219.6	167	4290
9	367	209.4	161	4290
10	389	181.5	169	4290
11	421	179.7	179	7890
12	471	221.3	149	7890
13	469	209.3	168	7890
14	511	241.3	159	7890
15	529	247.8	162	7890
16	532	201.4	152	7890
17	549	251.3	152	7890
18	567	249.3	189	7890
19	597	251.3	191	9200
20	643	269.4	169	9200

Note: The first 16 groups of characteristic index data are used to train the neural network, and the last 4 groups are used to verify the feasibility of the training network.

**Table 3:** Training effect of ANN.

No	Precision loss (㎛)	Stiffness (N/㎛)	Intensity (MPa)	Remanufacturing cost(¥)	
				Actual value	Predictive value
1	108	241.3	135	1930	1870
2	229	183.1	132	4420	4290
3	427	171.2	175	8150	7890
4	611	254.9	189	9550	9200

Note: The remanufacturing cost can be predicted by inputting the characteristic index of the spindle to be tested. The relative error, which is defined as the difference between the predicted value and the actual value, should be within the allowable range. It is concluded that the training achieves the expected effect. Therefore, the complex functional relationship between the remanufacturing price and its influencing factors can be obtained using the ANN method through sample learning.

## Construction of the evaluation system

### System solution

According to the above-mentioned characteristics of the ECS, the advantages and disadvantages of the remanufacturing scheme are relative concepts so that a comprehensive evaluation is required in this regard. To make the weight indicator more reasonable, the method of combining subjective weights (analytic hierarchy process) and objective weight (entropy method) are used in the present study to determine the comprehensive weight of each indicator. Then an analysis matrix is established based on the improved fuzzy comprehensive evaluation method to evaluate different schemes.

**Fig 2:**
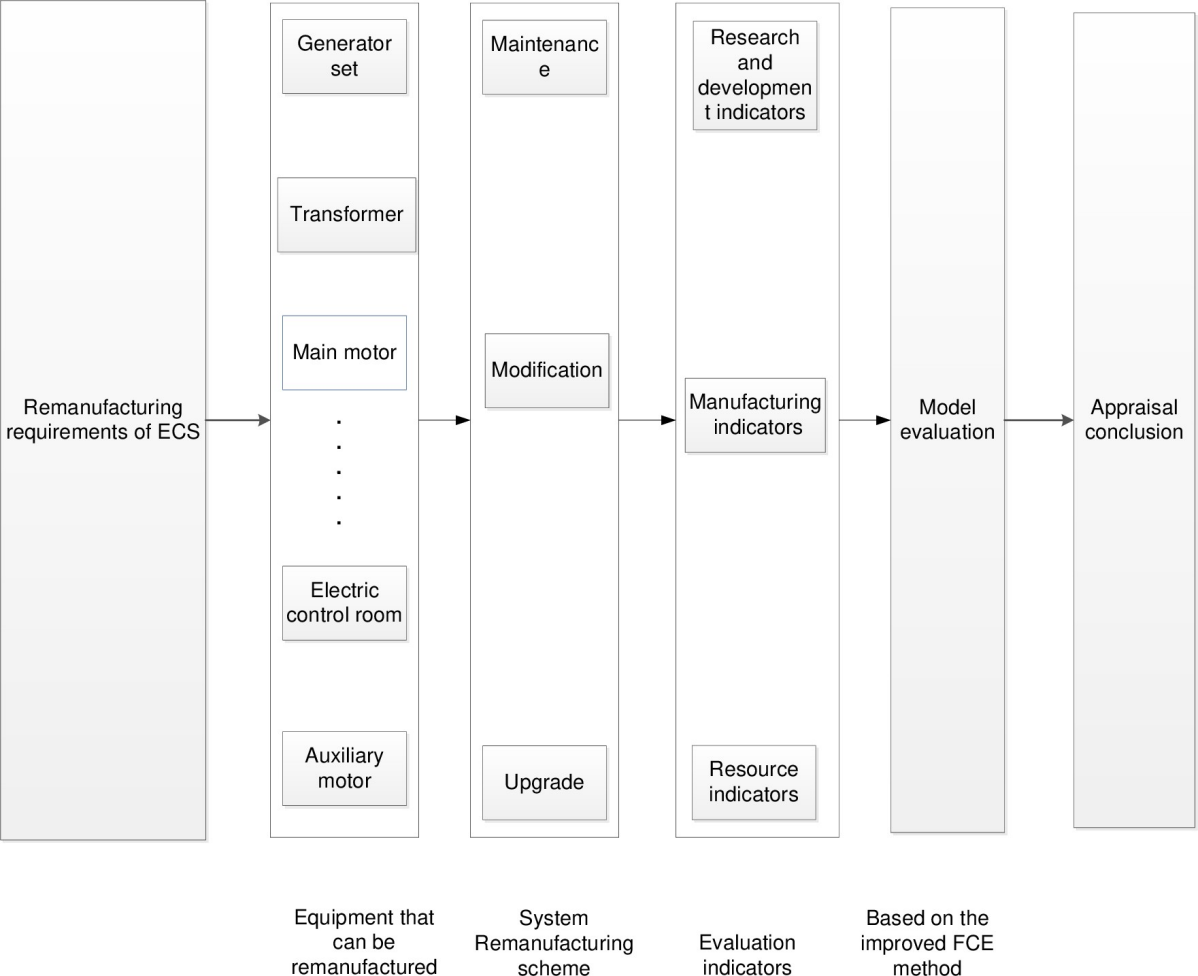
Flowchart of the proposed scheme.

In the present study, the following construction scheme is proposed in Fig 2:

For a certain AC/DC ECS, the system is decomposed first. For each component, all remanufacturing equipment and transformation schemes are listed and then classified according to the remanufacturing parts classification method. Based on the ANN cost prediction model, the remanufacturing cost of some equipment (except for the first type) is evaluated, and the remanufacturing plan is formulated.

Based on the analytic hierarchy process (AHP) and entropy method, three first-level indicators and six second-level indicators are defined to realize the preliminary screening of the components. Simultaneously, the evaluation comprehensive weight indicators are calculated.

Then the evaluation model and analysis matrix is designed based on the improved fuzzy comprehensive evaluation method, and the scoring and grading system is built based on the general characteristics of the project and special requirements of users.

When the system remanufacturing target is given, the evaluation model and composite weight of indicators can be constructed to determine the score and hierarchy of different schemes, thereby guiding experts to put forward the target improvement suggestions to complete the user decision support.

### Principles of the evaluation system construction

When the ECS is evaluated, it is necessary to determine the corresponding evaluation indicators and calculate the comprehensive evaluation weight indicators. Evaluation indicators should meet the requirements of design, development, manufacturing, and resource [13]. It is worth noting that the evaluation principles of ECS remanufacturing can be summarized as the following:

1. Research and development indicators
  These indicators mainly reflect the cost of design, research, and development, and evaluate whether the design and process inputs for remanufacturing of products can bring benefits. These indicators are especially important for new and customized products. Generally, the lower these indicators, the more justifiable the scheme.
2. Manufacturing indicators
  These indicators point-blank and reprocessing costs, evaluate the production costs and remanufacturing investment, but do not cover the power consumption. Generally, these indicators should be reduced as much as possible.
3. Resource indicators
  These indicators mainly refer to the consumption of resources such as water, electricity, and gas, and the cost of pollutant treatment. Accordingly, these indicators should be as low as possible.
  Accordingly, the structure model of the analytic hierarchy process can be constructed.

#### Construction of the subjective weight

**Fig 3:**
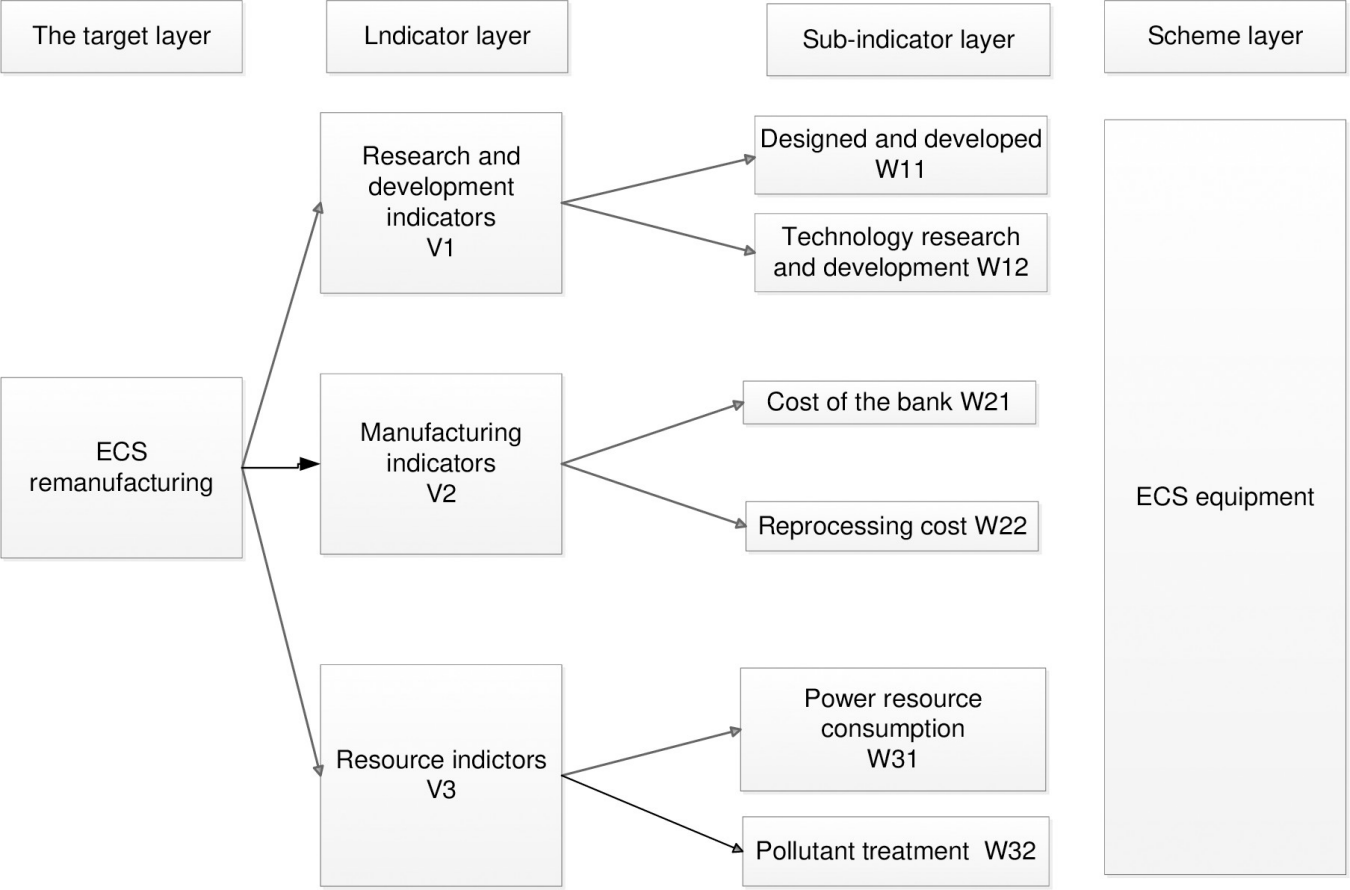
Structural diagram of the analytic hierarchy process.

In the present study, an analytic hierarchy process (AHP) is applied to construct the subjective weight. AHP is a systematic analysis method that decomposes the elements related to the decision-making into the target, indicator, scheme, and other levels and conducts a qualitative and quantitative analysis based on the expert scoring method. The main process of the AHP can be summarized as follows:

1. Constructing the indicator system, as shown in Fig 3;
2. Scoring each indicator, and introducing the 1–9 scale method, as shown in Table 4;
3. Normalizing and calculating the subjective weight vector;
4. Performing the consistency test;
5. Confirming the weight value W_i_.

**Table 4:** 1–9 Scale method table.

Scale	define	instructions
1	As important	The two vectors are equally important
3	A little important	The former is slightly more important than the latter
5	Obviously important	The former is obviously more important than the latter
7	More important than	The former is more strongly important than the latter
9	Extremely important	The former is more important than the latter
2,4,6,8	The median	The intermediate value of the above adjacent judgments
The bottom	The bottom	Two vectors are compared, the latter is more important than the former scale

#### Construction of the objective weight

In this section, the entropy method is applied to construct the objective weight. Entropy is an indicator reflecting uncertainty of the system state. Entropy can be used to measure the amount of information in the indicator data in the evaluation system and determine the weight of each indicator. Accordingly, the entropy theory can be considered an objective weight assignment method. Its main stages of the entropy method are as follows:

1. Constructing the original data matrix;
2. Constructing the normalized matrix;
3. Calculating the entropy value of the i^th^ indicator;
4. Calculating the objective weight (entropy weight) of the i^th^ indicator. To minimize the costs, the extremely small expression of the range transformation method is adopted in the entropy method:

$$x^{*}=\frac{\max-x}{\max-\min}$$ (1)

#### Construction of the compound weight

Although the analytic hierarchy process is based on expert opinion, it has subjective deviations. The indicator obtained by the entropy method may eliminate the subjective bias caused by human interference. However, due to the neglect of expert opinions, it may not be in good agreement with the actual situation. Therefore, the two methods are coupled to determine respective proportions. Accordingly, the comprehensive weight coefficient λ can be obtained from the following expression:

$$\lambda_{i}=kw_{i}+(1-k)u_{i}$$ (2)

Where K is the coupling coefficient, and 0≤ K ≤1. Based on the expert recommendation, this coefficient is set to K = 0.75.

#### Improved fuzzy comprehensive evaluation model

Combined with the characteristics of petroleum electronic control, a novel membership theory is proposed based on fuzzy mathematics to improve the evaluation model:

1. Selective computing model
  According to different fuzzy synthesis operations, there are generally four different calculation models of the fuzzy synthesis evaluation method^15^.
  Model ⅰ: Take large and small M (∧, ∨);
  Model ⅱ: The product is large M (•, ∨);
  Model ⅲ: Take a small upper bound sum M (∧, ⊕);
  Model ⅳ: Product summation type M (•, +).
  It should be indicated that models ⅰ, ⅱ, and ⅲ are applicable to cases where the main factors play a major role. Since multiple factors should be considered in the present study, model ⅳ is used in all calculations. This model can be mathematically expressed as follows:
$$b_{j}=\overset{n}{\underset{i=1}{\sum }}\lambda_{i}x_{i}^{T}),i=1,2,\cdots ,n$$ (3)
    Where λ_i_ denotes the composite weight, which is set according to the indicator, and x_i_^T^ is the expert scoring matrix corresponding to the indicator layer.
2. Grading system

In order to improve the fuzzy evaluation method, reduce the subjectivity of the experts and allow the user to understand the problem, the evaluation level should be classified considering the decision-making concepts. In this paper, project requirements can be divided into two categories, including "system uses" and "special requests", where the project contains six hierarchies and adopts the "classification" mode to quantify the ranks, as shown in Table 5.

**Table 5:** Description of the expert grading scale, x is the score given by experts.

item	Score interval	The hierarchy	Requirements
1	90≤x<100	A	Overall superior to project requirements
2	80≤x<90	B	Partial outperformance to project requirements (only one outperformance to requirements, and one to meet project requirements
3	70≤x<80	C	Two items only meet the requirements of the project
4	60≤x<70	D	The system meets the requirements of use, but it does not meet the special requirements of users. Minor modifications are required
5	50≤x<60	E	The system does not meet user requirements of use, but it meets the special requirements of users, which needs to be modified substantially
6	X < 50	F	The system does not meet the requirements of users

#### Analysis of comprehensive evaluation results

The final results of the above-mentioned calculations are compared with Table 5 to analyze improvement and optimization. If necessary, repeat the evaluation system construction process to meet the standard requirements.

## Case analysis

In order to verify the effectiveness of the method, the remanufacturing decision was applied to the maintenance of an ECS. The ECS was produced in 2007 and has been in service for more than 12 years. It was installed outdoor in a wet environment, subjected to perennial wind and rain. As a result, a large rusted area appeared on the painted surface. Then evaluating the remanufacturing solution is required after comprehensive testing. The system should meet the new standard after remanufacturing, and the extra budget cost should not exceed 22% of the newly purchased similar model. Based on the expert recommendation, the "improved fuzzy comprehensive evaluation method" is adopted to optimize several schemes, and then the validity of the method is verified by independent third-party data.

According to the characteristics of the ECS, early detection, sampling analysis, and expert recommendation, three feasible solutions are proposed: equipment only restores the original performance of remanufacturing (M1), part of the equipment modification + partial equipment restores the original performance of remanufacturing (M2), part of the equipment upgrade + part of the equipment restores the original performance of remanufacturing (M3).

### Classification of remanufactured parts

After testing the equipment on-site using the remanufactured parts classification method, it is found that parts of four equipment have remanufacturing value, then the performance degradation characteristics of related equipment parts are introduced into the remanufacturing cost prediction model to predict the remanufacturing cost. These parts are engine parts and fuel system of the generator set, "Rotor and stator" of the main motor, electric control room "room body structure”, and "radiator fin" of the transformer.

#### Construction of the subjective weight

Based on Fig 3, Table 4, and the construction process of the subjective weight of the ANALYTIC hierarchy process, the weights are calculated and the obtained results are presented in Table 6. The comparison of indicators indicates that the "R&D indicator" occupies the highest proportion in the subjective weight and is the most critical factor.

**Table 6:** Analysis matrix of an electrical control system scheme (subjective).

Weight of sub-indicator layer relative to indicator layer						
	W11	W12	W21	W22	W31	W32
M1 Scheme	0.67	0.33	0.3	0.7	0.6	0.4
	λ_max_ = 2, CI = 0, RI = 0		λ_max_ = 2, CI = 0, RI = 0		λ_max_ = 2, CI = 0, RI = 0	
M2 Scheme	0.7	0.3	0.4	0.6	0.55	0.45
	λmax = 2, CI = 0, RI = 0		λmax = 2, CI = 0, RI = 0		λmax = 2, CI = 0, RI = 0	
M3 Scheme	0.75	0.25	0.25	0.75	0.6	0.4
	λmax = 2, CI = 0, RI = 0		λmax = 2, CI = 0, RI = 0		λmax = 2, CI = 0, RI = 0	
Weight of indicator layer relative to the target layer						
	V1		V2		V3	
M1 Scheme	0.531		0.322		0.147	
	λ_max_ = 2,CI = 0, RI = 0.25, CR = 0.018<0.1					
M2 Scheme	0.595		0.277		0.128	
	λ_max_ = 3.01, CI = 0, RI = 0.25, CR = 0.011<0.1					
M3 Scheme	0.648		0.23		0.122	
	λ_max_ = 3, CI = 0, RI = 0.25, CR = 0.007<0.1					

### Construction of the objective weight

In this section, the cost of four equipment under different indicators is used as an objective indicator in Eq (1). The obtained weight values of the three schemes are shown in Table 7. It is observed that the "resource indicator" accounts for the highest proportion of the objective weight, which is the most critical factor.

**Table 7:** Analysis matrix of the ECS scheme (objective).

Weight of sub-indicator layer relative to indicator layer			
Evaluation indicators	M1 scheme weight	M2 scheme weight	M3 scheme weight
Design and develop W11	0.11766	0.11766	0.11766
Process development W12	0.11766	0.11768	0.11766
Blank cost W21	0.10867	0.10867	0.10867
Reprocessing cost W22	0.10838	0.10838	0.10838
Power resource consumption W31	0.28063	0.28062	0.28063
Contaminant treatment W32	0.26699	0.26699	0.26699
Weight of indicator layer relative to the target layer			
Evaluation indicators	M1 scheme weight	M2 scheme weight	M3 scheme weight
R&D indicator V1	0.23548	0.23595	0.23549
Manufacturing indicator V2	0.21708	0.21749	0.21704
Resource Indicator V3	0.54744	0.54656	0.54747

#### Construction of the compound weight

According to the weight values in Eq (2) and Tables 6 and 7, the composite weight can be obtained. The obtained results in this regard are presented in Table 8. It is found that the proportion of the "R&D indicator" is still the highest. Accordingly, it is determined as the most critical factor.

**Table 8:** Compound weight matrix.

Weight of sub-indicator layer relative to indicator layer			
Evaluation indicators	M1 scheme compound weight	M2 scheme compound weight	M3 scheme compound weight
Design and develop W11	0.5319	0.5544	0.5919
Process development W12	0.2769	0.2544	0.2169
Blank cost W21	0.2522	0.3272	0.2147
Reprocessing cost W22	0.5521	0.4771	0.5896
Power resource consumption W31	0.5202	0.4827	0.5202
Contaminant treatment W32	0.3667	0.4042	0.3667
Weight of indicator layer relative to the target layer			
Evaluation indicators	M1 scheme compound weight	M2 scheme compound weight	M3 scheme compound weight
R&D indicator V1	0.4571	0.5052	0.5449
Manufacturing indicator V2	0.2958	0.2621	0.2268
Resource Indicator V3	0.2471	0.2326	0.2284

#### Improved fuzzy comprehensive evaluation model

The comprehensive evaluation model is constructed according to Eq (3) and Table 8. The obtained results in this regard are shown in Table 9.

**Table 9:** Comprehensive evaluation table.

Expert score of sub-indicator layer			
Evaluation indicators	M1 scheme score	M2 scheme score	M3 scheme score
Design and develop W11	98	80	75
Process development W12	96	75	63
Blank cost W21	75	69	61
Reprocessing cost W22	69	55	50
Power resource consumption W31	93	75	95
Contaminant treatment W32	95	73	97
Expert score of the indicator layer			
Evaluation indicators	M1 scheme score	M2 scheme score	M3 scheme score
R&D indicator V1	78.71	63.43	58.06
Manufacturing indicator V2	57.01	48.81	42.57
Resource Indicator V3	83.22	65.71	84.99
Expert score of the target layer			
Evaluation indicators	M1 scheme score	M2 scheme score	M3 scheme score
ECS prolongs life	73.41	60.13	60.70
The hierarchy	C	D	D

Tables 8 and 9 show the ranking and analysis of different remanufacturing schemes, which provide a basis for users to make decisions.

1. R&D indicator score: SW1 = W1R1 = (78.71,63.43,58.06)
  Sorting result: M1 scheme > M2 scheme > M3 scheme.
  At the LEVEL of RESEARCH and development, THE M1 scheme has the lowest input cost and the highest score. This may be attributed to the lowest momentum change; In terms of "process research and development", the M3 program mainly focuses on transformation and upgrading, so the highest investment cost and the lowest score occur.
2. Manufacturing indicator score: SW2 = W2R2 = (57.01,48.81,42.57)
  Sorting result: M1 scheme > M2 scheme > M3 scheme.
  Manufacturing level: In the M1 scheme, the existing blank is used for processing, so the "blank cost" is the lowest; In terms of "reprocessing cost", the largest workload occurs in the M3 scheme, which may be attributed to the overall outsourcing and many interfaces that should be transformed.
3. Resource indicator score: SW3 = W3R3 = (83.22,65.71,84.99)
  Sorting result: M3 scheme > M1 scheme > M2 scheme.
  Resource level: M3 scheme is mainly outsourced, so the consumption of "power resources" is the lowest; Since both remanufacturing and retrofitting are considered in the M2 scheme, it can be applied to handle the largest amount of pollutants.
4. Comprehensive evaluation indicator score: SV = WVRV = (73.41,60.13,60.7)
  Sorting result: M1 scheme > M3 scheme > M2 scheme.
  Considering the impact of the indicators at the above three levels, M1 has obvious advantages in R&D and resources. Table 7 shows that the M1 scheme of classes C, M2, and M3 belong to D. This may be attributed to the involvement of system transformation (M2 and M3) of multiple devices in the system transformation, and transformation process involving research and development, manufacturing and resources. In order to ensure the overall efficient operation of ECS, and prevent energy waste caused by equipment power mismatch in the next process, it is essential to investigate and develop the concepts of "design", "technological R&D", "blank cost" and "extra money on the rework costs", and improve the accumulated value of the requirements of users. The conclusion is consistent with the user-independent third-party appraisal institution, thereby confirming the expert opinion. Meanwhile, the end-user selected M1, thereby validating the evaluation system.

## Conclusions

Based on the ANN theory, fuzzy theory, and comprehensive evaluation method, an improved fuzzy evaluation method was proposed to evaluate various remanufacturing schemes of the ECS. In this regard, compositions and internal correlation characteristics of ECS were considered in the calculations. The main achievements of the present study can be summarized as follows:

1. Based on the system observation, the electronic remanufacturing evaluation system was constructed. It was found that the evaluation indicators cover a comprehensive range, and the evaluation conclusions approach the actual situation.
2. From the perspective of remanufacturing profit and overhaul cycle frequency of equipment parts, the remanufacturing cost forecasting model was constructed using the ANN. This method increases the classification speed, thereby reducing the classification time.
3. The feasibility of the proposed method is verified by the practical application of this method in a certain project. The ranking result of a comprehensive evaluation is M1 scheme > M3 scheme > M2 scheme. Considering the impact of the above three indicators, M1 has obvious advantages in R&D and resources. Moreover, the proposed decision-making method has good stability and can be adjusted according to the actual needs.
4. The weight of the composite indicator in the evaluation system shows the importance of each indicator, and the calculation results show that the "R&D indicator" is the most critical index in the evaluation system of ECS remanufacturing. Therefore, it is suggested to increase the investment in the field of research and development and reduce the overall cost in the later period.
